# Configurable pattern-based evolutionary biclustering of gene expression data

**DOI:** 10.1186/1748-7188-8-4

**Published:** 2013-02-23

**Authors:** Beatriz Pontes, Raúl Giráldez, Jesús S Aguilar-Ruiz

**Affiliations:** 1 Department of Computer Languages, University of Seville, Seville, Spain; 2 School of Engineering, Pablo de Olavide University, Seville, Spain

**Keywords:** Gene expression data analysis, Shifting and scaling expression patterns, Evolutionary biclustering

## Abstract

**Background:**

Biclustering algorithms for microarray data aim at discovering functionally related gene sets under different subsets of experimental conditions. Due to the problem complexity and the characteristics of microarray datasets, heuristic searches are usually used instead of exhaustive algorithms. Also, the comparison among different techniques is still a challenge. The obtained results vary in relevant features such as the number of genes or conditions, which makes it difficult to carry out a fair comparison. Moreover, existing approaches do not allow the user to specify any preferences on these properties.

**Results:**

Here, we present the first biclustering algorithm in which it is possible to particularize several biclusters features in terms of different objectives. This can be done by tuning the specified features in the algorithm or also by incorporating new objectives into the search. Furthermore, our approach bases the bicluster evaluation in the use of expression patterns, being able to recognize both shifting and scaling patterns either simultaneously or not. Evolutionary computation has been chosen as the search strategy, naming thus our proposal Evo-Bexpa (**Evo**lutionary **B**iclustering based in **Ex**pression **Pa**tterns).

**Conclusions:**

We have conducted experiments on both synthetic and real datasets demonstrating Evo-Bexpa abilities to obtain meaningful biclusters. Synthetic experiments have been designed in order to compare Evo-Bexpa performance with other approaches when looking for perfect patterns. Experiments with four different real datasets also confirm the proper performing of our algorithm, whose results have been biologically validated through Gene Ontology.

## Background

DNA microarray technologies are used to analyse the expression level of many genes in a single reaction quickly and in an efficient manner. Different types of microarray chips have been designed for different investigations, being expression chips the most common application. They are used to determine the expression patterns of genes that correspond to different samples, where the samples may vary according to experimental conditions and/or physiological states. They may even be extracted from different individuals, tissues or developmental stages [1]. The applications of this kind of microarrays involve to determine gene functions, find new genes, study gene regulation and assess how they have evolved over time.

The raw data of a microarray experiment is an image, in which the colours and intensities reflect the expression level of each gene and each sample. This image is processed in order to obtain a numerical gene expression matrix, in which rows correspond to the genes under study and the columns refers the different samples. A special characteristic of these expression matrices is that they are very unbalanced, in the sense that the number of genes is much larger (usually thousands of genes) than the number of samples (usually less than a hundred) [2]. Therefore, analysing these kind of matrices implies understanding the relationships of a space of lots of variables (genes) from only a few measured points (experimental conditions).

In order to obtain relevant knowledge from microarray data, similarities among genes and samples need to be carried out in many different ways, depending on the specific application. Due to the complexity of these tasks, together with the huge amount of data, diverse data mining and machine learning approaches have been studied to produce a great variety of software for the analysis of gene expression data from microarrays.

### Gene expression microarray analysis

Focussing the expression matrices analysis on the genes, one of the most studied goals is to extract information on how gene expression patterns vary among the different samples, finding groups of co-expressed genes. If two different genes show similar expression patterns across the samples, this suggests a common pattern of regulation, possibly reflecting some kind of interaction or relationship between their functions [3].

Within data mining techniques it is possible to differentiate two main sets of algorithms, depending on the use (supervised learning) or not (non-supervised learning) of previous knowledge on the data. Classification has been extensively studied within gene expression data as a supervised technique [4-8], where labelled data is used to create an algorithm able to assign any new input data to its proper class.

On the other hand, non-supervised learning is used when no previous assignations are available; the goal is to divide the data into clusters of samples and to identify the differences between the genes that characterize such groups. The application of clustering techniques to gene expression data has also been broadly studied in the literature [9-11]. Nevertheless, there exists two main restrictions in the use of clustering algorithms: (1) genes are grouped together according to their expression patters across the whole set of samples, and (2) each gene must be clustered into exactly one group. This last limitation is two-fold: firstly, it means that a certain gene cannot be present in different groups, thus forbidding overlapping among clusters; secondly, it confines each gene to be a member of any cluster, even if it is not co-regulated with any of the other genes in the cluster.

However, genes might be relevant only for a subset of samples. This is essential for numerous biological problems, such as the analysis of genes contributing to certain diseases, assigning biological functionalities to genes or when the conditions of a microarray are diverse [12]. Thus, clustering should be performed on the two dimensions (genes and conditions) simultaneously. Also, many genes may be grouped into diverse clusters (or none of them) depending on their participation in different biological processes within the cell [13]. These characteristics are covered by biclustering techniques, which have also been largely applied to microarray data [14-16]. The groups of genes and samples found by biclustering approaches are called biclusters.

Finding significant biclusters in a microarray is a much more complex problem than clustering [17]. In fact, it has been proven to be a NP-hard problem [18]. Consequently, the majority of the proposed techniques are based on optimization procedures as the search heuristics. The development of both a suitable heuristic and a good cost function for guiding the search is essential for discovering interesting biclusters in an expression matrix. Furthermore, having a suitable evaluation measure for biclusters is important as it can be used for comparing the performances of different biclustering approaches, which is an unsolved task nowadays.

In order to design an effective evaluation measure for biclusters, we have focused our research on the study of different types of expression patterns in the literature.

### Gene expression patterns in biclusters

Several types of biclusters have been described and categorized in the literature, depending on the pattern exhibited by the genes across the experimental conditions [19]. For some of them it is possible to represent the values in the bicluster using a formal equation. In the following, let ℬ be a bicluster consisting in a set *I* of |*I*| genes and a set *J* of |*J*| conditions, in which *b*_*ij*_ refers to the expression level of gene i under sample j. 

•**Constant values:***b*_*ij*_=*Π*

•**Constant values on rows or columns**

–Additive: *b*_*ij*_=*Π*+*β*_*i*_, *b*_*ij*_=*Π*+*β*_*j*_

–Multiplicative: *b*_*ij*_=*Π*×*α*_*i*_, *b*_*ij*_=*Π*×*α*_*j*_

•**Coherent values on both rows and columns**

–Additive: *b*_*ij*_=*Π*+*β*_*i*_+*β*_*j*_

–Multiplicative: *b*_*ij*_=*Π*×*α*_*i*_×*α*_*j*_

where *Π* represents any constant value for ℬ, *β*_*i*_(1≤*i*≤|*I*|) and *β*_*j*_(1≤*j*≤|*J*|) refer to constant values used in additive models for each gene *i* or condition *j*; and *α*_*i*_,(1≤*i*≤|*I*|) and *α*_*j*_,(1≤*j*≤|*J*|) correspond to constant values used in multiplicative models for each gene *i* or experimental condition *j*.

Other kind of biclusters correspond to those in which their values exhibit **coherent evolutions**, thus showing an evidence that the subset of genes is up-regulated or down-regulated across the subset of conditions without taking into account their actual expression values. In this situation, data in the bicluster cannot be represented by any mathematical model.

The most general situation that can be described using a mathematical formula is when a bicluster has coherent values on both rows an columns, for the additive and multiplicative model at the same time. When it is the case, it is said that the bicluster follow a perfect shifting and scaling pattern, and its values can be represented by this equation:

(1) $$b_{\text{ij}}=\Pi_{i}\times \alpha_{j}+\beta_{j},$$

where *Π*_*i*_(1≤*i*≤|*I*|) refer to constant values for each gene/row *i*. Since 2000, several quality measures for biclusters have been proposed together with different heuristics. Nevertheless, to the best of our knowledge, none of the former proposed quality measures is able to recognize a perfect shifting and scaling pattern in a bicluster. Nevertheless, this is the most general situation and also the most probable when working with gene expression data. In this sense, we have recently developed a standardization-based procedure for assessing biclusters quality. This measure has been named VE ^*t*^ and has been proven to be effective for recognizing both types of patterns simultaneously in biclusters (see section *Biclusters Evaluation in Evo-Bexpa*).

### Biclustering approaches based on evaluation measures

#### Mean squared residue

Cheng and Church [20] were the first in applying biclustering to gene expression data. They introduced one of the most popular biclustering algorithms that combines a greedy search heuristic for finding biclusters with a measure for assessing the quality of such biclusters.

The original algorithm of Cheng and Church (henceforth CC) adopts a sequential covering algorithm in order to return a list of *n* biclusters from an expression data matrix. In order to assess the quality of biclusters the algorithm uses the *Mean Squared Residue* (MSR). This measure aims at evaluating the coherence of the genes and conditions of a bicluster *B* consisting of *I* rows and *J* columns. MSR is defined as:

(2) $$\text{MSR}(B)=\frac{1}{\left|I|\cdot |J\right|}\overset{\left|I\right|}{\underset{i=1}{\sum }}\overset{\left|J\right|}{\underset{j=1}{\sum }}{\left(b_{\text{ij}}-b_{\text{iJ}}-b_{\text{Ij}}+b_{\text{IJ}}\right)}^{2},$$

where *b*_*ij*_, *b*_*ij*_, *b*_*ij*_ and *b*_*ij*_ represent the element in the *i*^*th*^ row and *j*^*th*^ column, the row and column means, and the mean of *B*, respectively. The lower the mean squared residue, the stronger the coherence exhibited by the bicluster, and the better its quality. If a bicluster has a mean squared residue lower than a given value *δ*, then it is called a *δ*-bicluster. If a bicluster has MSR equal to zero, it means that its genes fluctuate in exactly the same way under the subset of experimental conditions, and thus it can be considered a perfect bicluster.

Nevertheless, MSR has been proven to be inefficient for finding certain types of biclusters in microarray data, especially when they present strong scaling tendencies [21]. In fact, MSR is only able to capture shifting tendencies within the data [22]. Furthermore, CC also presents some other disadvantages due to the search strategy and the use of a threshold for rejecting solutions, since this threshold is dependent on each database and has to be computed before applying the algorithm [23].

In spite of the MSR constraints, it has been widely used in many proposals. These proposals are based on a diverse range of heuristics: greedy search [24], genetic algorithms (both single and multi-objective) [17,25], simulated annealing [26], and fuzzy biclustering, among others. More recently, MSR has also been incorporated as cost function in multiobjective heuristics based on Particle Swarm Optimization [27], Artificial Immune Systems [28], and in a variant of the GRASP approach [29].

#### Scaling mean squared residue

Mukhopadhyay et al. [30] have recently developed an evaluation measure for biclusters which is able to recognize scaling patterns. In their work, they analyse the reasons why MSR is able to recognise shifting patterns in biclusters but no scaling patters. Using the mathematical formula for scaling patterns, they define a metric which is then proved to identify scaling patterns. This new measure is named SMSR, from Scaling MSR, and it is shown in equation 3. Nevertheless, SMSR is not capable of identifying shifting patterns.

(3) $$\text{SMSR}(B)=\frac{1}{\left|I|\cdot |J\right|}\overset{i=|I|}{\underset{i=1}{\sum }}\overset{j=|J|}{\underset{j=1}{\sum }}\frac{{\left(b_{\text{iJ}}\times b_{\text{Ij}}-b_{\text{ij}}\times b_{\text{IJ}}\right)}^{2}}{b_{\text{iJ}}^{2}\times b_{\text{Ij}}^{2}}$$

SMSR has been incorporated into a greedy search strategy similar to that of Cheng and Church. This methodology, therefore, shares the same disadvantages with CC, and it is also necessary to stablish a limit value for SMSR for each database. In order to also find biclusters with shifting patterns, the authors propose an adapted algorithm in which CC algorithm is applied twice, the first time using MSR as evaluation measure and the second time using SMSR. This allows to find biclusters with shifting patterns and also biclusters with scaling patterns, but it does not find biclustering with both kind of patterns simultaneously.

#### HARP Algorithm

Yip et al. [31] presented a biclustering approach named HARP (Hierarchical approach with Automatic Relevant dimension selection for Projected clustering) based on projected clustering. They also introduced an evaluation metric slightly different from MSR, in which the quality of a bicluster is measured as the sum of the relevance indices of the columns. Relevance index *R*_*Ij*_ for column *j*∈*J* is defined as

(4) $$R_{\text{Ij}}=1-\frac{\sigma_{\text{Ij}}^{2}}{\sigma_{\mathrm{.j}}^{2}},$$

where $\sigma_{\text{Ij}}^{2}$ (local variance) and $\sigma_{\mathrm{.j}}^{2}$ (global variance) are the variance of the values in column *j* for the bicluster and the whole data set, respectively. Note that the relevance index for a column is maximized if its local variance is zero, provided that the global variance is not. Based on this relevance index, the quality of a cluster is measured as the sum of the index values of all the selected conditions.

At the beginning of the algorithm there are as many biclusters as genes. The process consist in iteratively merging biclusters until a certain criterion is met, choosing those experimental conditions that satisfy a specific threshold requirements, taking into account the relevance indexes. Optionally, a re-assignation procedure is applied, where biclusters with very few elements are removed and elements are assigned to the closest bicluster, according to a distance measure.

This algorithm presents several drawbacks, being the most important one the kind of biclusters it is capable to find. Due to the nature of their evaluation measures, the only bicluster patterns that maximize the quality are constant biclusters (either on rows or on columns). Furthermore, the way in which the algorithm works does not allow overlapped elements among biclusters, which is one of the most important differences between clustering and biclustering methodologies.

#### Virtual error

The basic idea behind the Virtual Error (VE) [32] is to measure how genes in a bicluster follow the general tendency within the group. In order to catch the general tendency of the genes across the conditions contained in the bicluster, a new gene (the so-called *virtual gene*) is computed as the mean of all the genes in the bicluster. This way, this virtual gene symbolizes the common tendency of the set of genes for the given bicluster.

VE aims at measuring the extend to which all the genes in the biclusters resemble the virtual gene. In order to carry out a fair comparison in terms of shifting and scaling patterns, a process of gene standardization is perform on all genes, including the virtual one. This way, genes values are scaled to a common range. After that, VE is defined as the average value of all the differences between the standardized expression values of the bicluster and the standardized virtual gene. The more similar the genes are, the lower the value for VE. In fact, VE has proven to be 0 for those biclusters presenting either shifting or scaling patterns [32]. It has also been proven that when the data in a bicluster resembles a perfect pattern but containing some other noise data, VE will have a greater value depending on the amount of noise data in the bicluster [33]. VE has been used in an evolutionary search strategy producing satisfactory results and improving those obtained with other evaluation measures [32], being able to recognized both shifting and scaling patters, though no simultaneously.

### Non metric-based biclustering algorithms

Not all existing biclustering approaches base their search for biclusters on evaluation measures. There exists a diverse set of biclustering tools that follow different strategies and algorithmic concepts which guide the search towards meaningful results. Among others, most popular algorithms include Ben-Dor et al. [34] approach’s Order Preserving Sub Matrix (OPSM) algorithm, which tries to identify large submatrices in which the expression levels of all genes induce the same linear ordering of the samples. Iterative Signature Algorithm (ISA) was proposed by J. Ihmels et al. [35,36] and applies the signature algorithm in order to find transcription modules, which are self-consistent regulatory unit consisting of a set of co-regulated genes and the experimental conditions that induce their co-regulation. Murali and Kasif [37] proposed the use of xMOTIFs (conserved gene expression Motifs) for the representation of gene expression data. Their algorithm looks for large xMOTIFs in which genes are expressed in the same state across all samples in it. Finally, Bimax has been presented by Prelic et al. as a fast divide-and-conquer algorithm capable of finding all maximal bicliques in a corresponding graph-based matrix representation [38].

Prelic et al. also developed a Biclustering Analysis Toolbox (BicAT) [39] which includes implementations of Bimax and also the other three algorithms (OPSM, ISA and xMOTIFs), together with CC (see section *Biclustering approaches based on Evaluation Measures*). In this work we have compared the results of our approach on both synthetic and real data sets with these five different approaches.

More recently, QUBIC has been presented as a qualitative biclustering algorithm, in which the input data matrix is first represented as a matrix of integer values. Afterwards the algorithm looks for genes with identical integer values across a subset of conditions [40]. Hochreiter et al. [41] have developed a generative multiplicative model for the biclustering problem, assuming realistic non-Gaussian signal distributions with heavy tails. They also assumed gene expression data to be preprocessed and filtered. Hierarchical clustering has also been used by Huang et al. [42], incorporating a sub-dimensional search strategy in an effort to reduce the search space dimension, while Sill et al. [43] have incorporated stability selection to improve a sparse singular value decomposition (SSVD) approach. Other works are based on a previous binarization of the data, such as DeBi [44]. After binarizing the data, DeBi consist of three stages for finding, extending and filtering seed bicluters. Although no evaluation measure for biclusters is defined, Fisher exact text is used in the extending phase.

## Methods

This section details our biclustering approach, consisting in a sequential covering method [45], where the function that obtains each bicluster is an evolutionary algorithm. Our algorithm has been named **Evo-Bexpa**, from **Evo**lutionary **B**iclustering based in **Ex**pression **Pa**tterns. The evolutionary process inside Evo-Bexpa consists of a genetic algorithm guided by a fitness functions in which several objectives are taken into account. These objectives are easily configurable, with the possibility of specifying user preferences on some characteristics of the results, specifically the number of genes, number of conditions, overlapping amount and gene mean variances. This way, if any previous information related to the microarray under study is available, the search can be guided towards the preferred types of biclusters. Furthermore, other objectives can also be easily incorporated into the search, as well as any objective may be ignored by setting its weight to zero.

The problem of finding a single bicluster according to several objectives corresponds to a multi-objective optimization problem, in which two or more conflicting objectives need to be optimized. The strategy of constructing a single Aggregate Objective Function (AOF) has been adopted in order to solve this multi-objective problem ([46]). This way, it is possible to specify the relative influence of each objective in the bicluster evaluation, allowing thus our algorithm to be configurable.

In the following subsections we first explain the different objectives taken into account in the bicluster evaluation. Afterwards, the evolutionary algorithm behind Evo-Bexpa is depicted, including the initialization of the population, the generational change and also the way in which the different objectives have been combined to form the fitness function.

### Biclusters evaluation in Evo-Bexpa

This subsection details the biclusters characteristics taken into account in their evaluation. In our approach we have individualised four different objectives, attending to the extent to which a bicluster follow a perfect correlation pattern, its size, overlapping amount among different solutions and mean gene variance. This objectives have been chosen corresponding to the whole set of objectives used in different biclustering approaches in the literature, with independence of their applications.

#### VE^*t*^ for correlated pattern recognition

Transposed Virtual Error (VE ^*t*^) [33] has been used as the quality measure for biclusters, being one of the most important objectives in the fitness. It is based on the concepts of expression patterns and quantifies the degree of correlation among genes in a bicluster. VE ^*t*^ is computed similarly to VE (see section *Biclustering approaches based on Evaluation Measures*) but in the transposed way. The first step is the creation of a *Virtual Condition*, which is a vector containing the means of every row in the bicluster, as represented in equation 5. This virtual condition *ρ* will have, therefore, as many elements as genes are contained in the bicluster.

(5) $$\rho_{i}=\frac{1}{\left|J\right|}\overset{\left|J\right|}{\underset{j=1}{\sum }}b_{\text{ij}}$$

Afterwards, a process of standardization is carried out on both the bicluster data and the virtual condition, as depicted in equation 6, where $\sigma_{c_{j}}$ and $\mu_{c_{j}}$ represent the standard deviation and the arithmetic average of all the expression values for condition *j*, respectively. *μ*_*ρ*_ and *σ*_*ρ*_ also refer to the average and the deviation of the values of the virtual condition, respectively.

(6) $$\hat{b_{\text{ij}}}=\frac{b_{\text{ij}}-\mu_{c_{j}}}{\sigma_{c_{j}}},\hat{\rho_{i}}=\frac{\rho_{i}-\mu_{\rho }}{\sigma_{\rho }}$$

Finally, VE ^*t*^ measures the differences between the standardized values for every experimental condition and the standardized virtual condition, as in equation 7. Therefore, VE ^*t*^ is always positive, being its optimal value equals to 0.

(7) $$VE^{t}(ℬ)=\frac{1}{\left|I|\cdot |J\right|}\overset{\left|I\right|}{\underset{i=1}{\sum }}\overset{\left|J\right|}{\underset{j=1}{\sum }}(\hat{b_{\text{ij}}}-\hat{\rho_{i}})$$

VE ^*t*^ has been proven to be efficient to recognize both shifting and scaling patterns in biclusters either simultaneously or independently [33]. It has also been proven to present a linear increasing behaviour when the amount of error in a bicluster gets bigger, measured according to the distance from its nearest perfect pattern. When working with real data, it is very unlikely to find biclusters where VE ^*t*^ is equal to zero, due to the fact that although genes in a good bicluster share a common behaviour, it cannot be represented in an exact mathematical equation [47].

#### Bicluster volume

Bicluster volume is defined as the product of the number of genes and the number of samples. At this point we have two contrary objectives to be optimized. On the one hand, VE ^*t*^ has to be minimized and normally the smaller a bicluster is, the lower VE ^*t*^ will be. On the other hand, the volume has to be maximized and the general tendency is that bigger biclusters will have bigger values for VE ^*t*^. In order to design the volume term for the fitness we took into account the following issues: 

•Use of a *logarithmic scale*. Little changes in the number of rows or columns would not have a significant effect, depending on the bicluster size.

•*Two separated terms* for number of genes and conditions. This is necessary for avoiding too unbalanced biclusters, but also desirable in order to allow to configure each dimension size independently. Note that biclusters in which one dimension is very small are more probable to be nearer to a perfect pattern, and therefore, they have low VE ^*t*^ values. For this reason, it is preferable to optimize the size of both dimensions independently, thus avoiding obtaining biclusters made up of a great numbers of genes and only a few samples.

•*Fixed range*. The range of the values of the functions controlling both dimensions should not be dependant on any parameter value.

The final design of the term for the volume is the one shown in equation 8, where |*I*| and |*J*| refer to the number of genes and conditions, respectively, while *w*_*g*_ and *w*_*c*_ are the configuring parameters for both dimensions.

(8) $$\text{Vol}(ℬ)=(\frac{-\ln(|I|)}{\ln(|I|)+w_{g}})+(\frac{-\ln(|J|)}{\ln(|J|)+w_{c}})$$

**Figure 1:**
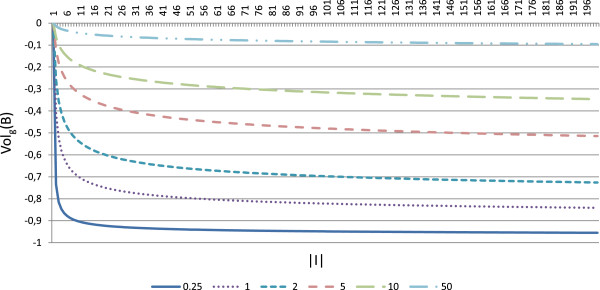
**Genes size term in volume evaluation.** This figure represent the term $\text{Vo}l_{g}(ℬ)=\frac{-\ln(|I|)}{\ln(|I|)+w_{g}}$ in equation 8, for different values of *w*_*g*_. For smaller values of *w*_*g*_, *Vol*_*g*_ decreases slower from a smaller value of the number of genes than for greater values of *w*_*g*_, implying that greater values of *w*_*g*_ should be selected in order to favour major number of genes.

Those terms whose constant value (*w*_*g*_ or *w*_*c*_) is greater decrease slower. Depending on the value of the constant used, the term will have more or less influence over the fitness function at the beginning of the algorithm, since initial biclusters are small and they grow along the evolutionary process. At a certain point, increasing the number of rows or columns for a certain solution would not compensate the lose of quality, according to the rest of objectives. The moment in which the algorithm stops increasing the size of the solutions and focuses on improving the quality depends on the value of the constants used. The smaller these constants are, the sooner the algorithm will stop increasing the size. Figure 1 represents the term for the number of genes in equation 8, for different values of the constant *w*_*g*_. It can be clearly seen that for the smaller value of *w*_*g*_ represented (*w*_*g*_=0.25), the function decreases slower from a smaller value of the number of genes than for greater values of *w*_*g*_.

Although we have found default values for the constants for both dimensions (rows and columns) that allow to obtain good solutions in every expression matrix we have tested, it is very easy to modify the fitness function in order to obtain solutions of different sizes if it is desirable. Increasing the constant associated to rows (*w*_*g*_) will produce biclusters with greater number of genes, while increasing the constant associated to columns (*w*_*c*_) will produce biclusters with more experimental conditions.

#### Overlapping among biclusters

Overlapping among biclusters is usually permitted but controlled in the literature [13]. Overlapping differs from VE ^*t*^ and volume in the sense that it cannot be evaluated on a bicluster by itself. Cheng and Church [20] try to avoid overlapping by replacing in the microarray data those values contained in each found bicluster with random ones. The main drawback of this strategy is that the replacement does not really avoid including those values in future biclusters. Therefore, if a bicluster is overlapped with a former one, that means that this new bicluster has been found using random values instead of the real ones.

In our work, we have adopted a strategy similar to the one used in [23], where a matrix of weights W the size of the microarray is initialized with zero values at the beginning of the algorithm. Every time a bicluster is found, the weight matrix is updated increasing by one those elements contained in the bicluster. In order to limit the overlap among biclusters, this matrix is used in the corresponding term of the fitness function as in equation 9, where *I* and *J* refers to the sets of rows and columns in the bicluster ℬ, respectively. *W*(*b*_*ij*_) corresponds to the weight of *b*_*ij*_ in W.

(9) $$\text{Overlap}(ℬ)=\frac{\underset{i\in I,j\in J}{\sum }W(b_{\text{ij}})}{\left|I|\times |J|\times (n_{b}-1\right)}$$

This term computes how many times the elements of ℬ have appeared in any former biclusters, and divides it by the size of ℬ and the order of the solution (*n*_*b*_) minus one. This way, we are being more permissive with the latest solutions, and also enclosing the overlapping factor in the interval [0,1].

#### Gene variance

Biclustering was first defined by Hartigan in 1972 [48], although it wasn’t applied to microarray data. The aim was to find a set of sub-matrices having zero variance, that is with constant values. Therefore, Hartigan used the variance of a bicluster to evaluate its quality. However, when working with gene expression data, it is preferable to obtain biclusters in which gene variances are high. This way, gene variance is used in biclustering of microarray data to avoid obtaining trivial biclusters, favouring those solutions in which genes exhibit high fluctuating trends. Gene variance of a bicluster is given by the mean of the variances of all the genes in it, as in equation 10.

(10) $$\text{GeneVar}(B)=\frac{1}{\left|I|\cdot |J\right|}\overset{\left|I\right|}{\underset{i=1}{\sum }}\overset{\left|J\right|}{\underset{j=1}{\sum }}{\left(b_{\text{ij}}-\mu_{g_{i}}\right)}^{2}$$

Existing biclustering approaches deal with gene variance in different ways. For instance, Cheng and Church [20] used a threshold value *δ* as an upper limit for their evaluation measure. This way, they search for biclusters with the maximum possible values for MSR below *δ*, rejecting thus trivial solutions in which there are no expression changes across the samples. Nevertheless, using such a limit presents a clear drawback, since *δ* has to be computed for each database before applying the algorithm (see section *Biclustering approaches based on Evaluation Measures*).

In our proposal, using VE ^*t*^ as a single objective would produce biclusters in which gene variance is considerable low. However, if VE ^*t*^ is combined with volume constraints favouring bigger solutions and overlapping control, the obtained results may not have so low variance. Despite this fact, we have also designed a term for controlling gene mean variance within the fitness function. This new term consists in the inverse of the gene mean variance in equation 10, since biclusters with higher gene variances are preferred and the fitness is going to be minimized in the algorithm.

### Evolutionary algorithm

Evo-Bexpa follows a sequential covering strategy, obtaining a single bicluster each time the evolutionary algorithm (Bexpa) is executed. Therefore, it has to be run *n* times if *n* biclusters are desired, where *n* is an user-defined parameter.

Genetic algorithms are classified as population-based meta-heuristics for combinatorial optimization, iteratively trying to improve several candidate solutions (population) with regard to a given measure of quality (fitness function) [49]. In contrast to other meta-heuristics descendent methodologies, such as simulated annealing [26], tabu search [50] or particle swarm optimization [51], genetic algorithms start with a set of possible solutions instead of a single one. This characteristic allows genetic algorithms to explore a larger subset of the whole space of solutions, at the same time as it helps them to avoid becoming trapped at a local optimum. These reasons make genetic algorithms very suited to the biclustering problem.

The first task when choosing a genetic algorithm for solving any problem is to decide an appropriate individual or chromosome representation for the possible solutions. We have adopted the same individual representation in other evolutionary biclustering works [52], where each bicluster is represented by a fixed sized binary string in which a bit is set to one if the corresponding gene or sample is present in the bicluster, and set to zero otherwise.

**Figure 2:**
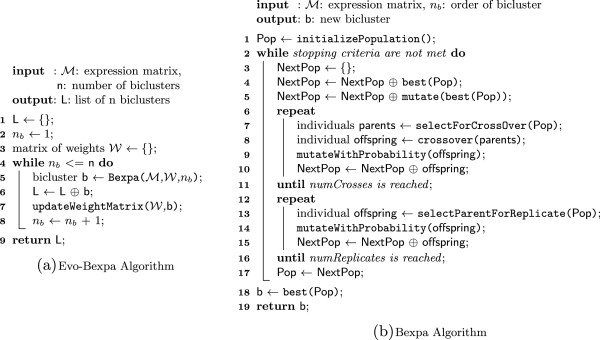
**Bexpa and Evo-Bexpa algorithms.** Evo-Bexpa (algorithm 1.a) consist in iteratively invoking Bexpa as many times as biclusters are desired (while loop in line 4). Starting with an empty list of biclusters (line 1), lines 6 to 8 represent the transition between two inner iterations, where the recently found bicluster by Bexpa (invoked in line 5) is stored into the result list, and also the matrix of overlapping weights is updated. This weight matrix was initialized with 0 values in line 3. The inner genetic algorithm (algorithm 1.b) searches for one bicluster at a time. The weight matrix W and the order of the next bicluster are given to Bexpa for evaluation purposes. Specifically, they are involved in the overlapping control process among different biclusters. Bexpa starts with the initialization of the population in line 1. Lines 2 to 17 correspond to the iterative process for the search of each solution.

Starting by an initial population, genetic algorithms select some individuals and recombine them to generate a new population of individuals. This process is repeated for a number of generations until the algorithm converges or certain criteria are met. Algorithms 1.a and 1.b in Figure 2 show the pseudo-codes of both the sequential (Evo-Bexpa) and genetic (Bexpa) strategies, respectively. Evo-Bexpa (algorithm 1.a) consist in iteratively invoking Bexpa as many times as biclusters are desired. Bexpa starts with the initialization of the population, followed by an iterative process for the search of a bicluster. Both stages will be detailed in the next subsections.

#### Initial population

Initial population procedure is essential in every evolutionary algorithm. Depending on the adopted strategy, the algorithm may converge to different solutions. Also, a suitable initial population strategy can even speed up the convergence [53].

Other evolutionary biclustering approaches have adopted a totally random initial population generation [54], where initial solutions are made up of a random number of elements (genes and samples) randomly chosen from the microarray, or also random strategies in which the chromosomes are made up of only one element (one gene and one condition) from the microarray [32]. In our experimental tests on synthetic data, we found that these kind of initializations did not give the algorithm an initial space solution good enough to come up to the best solution. Nevertheless, our algorithm always converged to the best solution when the initial population contained at least one 3×3 sized bicluster representing a partial solution. That is, this 3×3 sized bicluster is a sub-matrix of the solution. We used this kind of seeds since it represents the minimal partial solution in which correlation patterns are less probable to appear at random. Since 2×2 sized matrices of random values always present shifting and scaling behaviour, it would be impossible to differentiate their qualities.

Therefore, the initial strategy we have adopted consists in randomly generating individuals which represent 3×3 sub-matrices, henceforth seeds. The key is to generate much more seeds than the size of the population and then select the best ones. In fact, it is quite easy to compute the number of seeds needed to increase the probability that some of them are part of the solution, if it is known beforehand. The probability of a randomly generated seed to be part of the solution can be computed as the number of possible seeds in the solution divided by the number of possible seeds in the whole data matrix, as in equation 11, where M, N, |*I*| and |*J*| are the number of rows and columns of the microarray data matrix and the solution, respectively.

(11) Favorable_seedsTotal_seeds=|I|3×|J|3M3×N3

Thus, our algorithm computes the number or seeds needed in order to at least one of them is a part of the solution. This procedure can only be performed with synthetic data, but it also gives us an idea of the number of seeds to generate in the case of real data.

#### Generational change

Generational change is the mechanism that allows the population to improve its individuals, according to the fitness function and trying to converge to the optimal solution (lines 3 to 17 in Algorithm 1.b). For each generation, the new population is formed by incorporating individuals from the previous one in several ways: replicating themselves, being mutated, being crossed with other(s) individual(s) or combining some of these operators.

The next population in Bexpa is created by firstly adding the best individual of the current population to the next one, as it can be seen in line 4 of Algorithm 1.b in Figure 2. This process is called elitism and is usually applied in order to ensure the convergence of the algorithm [55]. Also, a mutated copy of the best individual is incorporated into the next population (line 5). The rest of individuals are generated by selecting one or two individuals and applying crossover or/and mutation. Selection is based on the use of the fitness function together with a random component. In our approach, we have used tournament of size 3 as selection mechanism [49]. 80% of the remaining individuals are generated by the crossover of two previously selected chromosomes (lines 6 to 10), while the other 20% individuals correspond to replications (lines 11 to 14). The resulting offspring is mutated with a certain probability in both cases.

Three distinct crossover operators are used in our algorithm with equal probability: one-point crossover, two-points crossover, and uniform crossover. We have also applied two different mutation operators: the simple and the uniform ones. The probability of the uniform mutator is much lower due to the fact that every position of the bit string is a candidate to be mutated in the uniform mutation.

The number of generations (iterations) has been set to 1500, although if there is no significant improvement after 150 consecutive generations, the execution is stopped. Crossover and replications percentages, as well as mutation probabilities and the number of generations have been set experimentally, although all of them are input parameters for the evolutionary algorithm and can be modified by the user.

#### Fitness function

The fitness function used in our algorithm for the evaluation of the potential solutions is presented here. Although we have used the four different objectives described above in our experiments, the fitness function is easily configurable by adding new objectives in the form of a mathematical formula.

In the context of evolutionary algorithms, the fitness function is a particular type of objective function used to summarise, as a single figure of merit, how close a given design solution is in order to achieve the set aims.

Equation 12 depicts the final fitness function used in our algorithm. Note that the goal is to minimize the value of every term, in order to find big-sized biclusters with a low value of VE ^*t*^, high gene variance and hardly overlapped.

(12) $$\begin{array}{l} \Phi (ℬ)=\frac{VE^{t}(ℬ)}{VE^{t}(ℳ)}+w_{s}\cdot \text{Vol}(ℬ)+w_{\text{ov}}\cdot \text{Overlap}(ℬ)\\ \;+w_{\text{var}}\cdot \frac{1}{1+\text{GeneVar}(ℬ)} \end{array}$$

Every term is weighted, except VE which acts as the reference objective. Nevertheless, the value of VE ^*t*^ for the bicluster has been divided by the VE ^*t*^ value of the whole microarray. This is due to the fact that the range of values of VE ^*t*^ depends of the values in each microarray. Although the algorithm pursuit to minimize it, the weight of the other terms of the fitness function would have to be recomputed when using a different microarray. In order to avoid this situation, we divide it by the VE ^*t*^ value of the whole microarray (ℳ refers to the microarray data matrix).

Modifying the weights associated to the different objectives leads the algorithm towards different kind of biclusters, according to their sizes, overlapping amount or gene variance. All weights have been designed in the same way; a lower value of a certain weight will result on biclusters with lower values for the corresponding characteristic, and vice versa. For example, a lower value of *w*_*s*_ will lead to small-sized biclusters, while bigger values of *w*_*s*_ will result on big-sized biclusters. In the results section we provide default values for every weight, which have been obtained experimentally and have produced meaningful results for all the databases under study. Also, we provide the user with a guidance on how the modification of the weights affect the different characteristic of the obtained biclusters.

Note that it is quite simple to add new objectives to the fitness. A new mathematical formula should be designed for each new bicluster feature to be taken into account. This formula will be minimized when inserted into the fitness function, and will also have a corresponding weight. In order to better control the effect on the results, it is preferable that the range of values were fixed, not dependant on the specific values of the microarray or bicluster.

## Results and discussion

This section presents a wide set of experiments performed to test the validity of Evo-Bexpa, both on synthetic and real data sets. The results have been compared with those obtained using five different approaches: OPSM [34], ISA [35,36], xMotifs [37], CC [20] and Bimax [38] (see section *Background* for a short description of each approach). All these five algorithms have been executed using BicAT ^*a*^ (Biclustering Analysis Toolbox)[39].

Next subsection presents an in-depth analysis on the performance of Evo-Bexpa when modifying the different configuration parameters introduced in *Bicluster Evaluation in Evo-Bexpa* section, as well as a study on the different parameters for the algorithms in BicAT. Later, subsections *Synthetic Data Experiments* and *Experiments on Real DataSets* describe the experiments carried out on artificial and real data sets, respectively.

### Analysis of parameters

Each biclustering approach needs different parameters to run. Although default parameters are provided which should guide the algorithms towards reasonable results, there is no detailed description on how their variations affect the obtained bicluster, for any of them. In this subsection we first describe the input parameters for each of the algorithms in BicAT (OPSM, ISA, xMotifs, CC and Bimax), trying to clarify the characteristic of the resulting biclusters affected by the modification of the different parameters. After that, we present a study on the parameter sensitivity for EvoBexpa.

#### Analysis of parameters for algorithms in BicAT

Bimax uses an underlying binary data model which assumes two possible expression levels per gene. Therefore, as a preprocessing phase, it is compulsory to discretize the expression values to binary values at a specific threshold and with a specific scheme. All values above the threshold will be set to one, all those below to zero. The discretization scheme defines if only down or up-regulated genes (or both) will be considered.

The algorithm also takes as input parameters the minimum number of genes and samples for the output biclusters. By specifying larger lower bounds, fewer biclusters will be returned, reducing thus the computing time. Default values for both the minimum number of genes and conditions have been set to two.

CC algorithm takes as input parameter two different thresholds, *δ* as the upper limit for MSR, which has already been mentioned, and *α*>1 as a threshold for the multiple node deletion phase. *δ* presents two main drawbacks: its value depends on the input microarray and has to be computed beforehand (there is no common default value), and also the use of *δ* blocks the algorithm from obtaining meaningful solutions [21,23]. Default value for *α* parameter has been set to 1.2, and the authors claim that when it is properly selected, the multiple node deletion phase is usually extremely fast. Nevertheless, there is no explanation on how does this value affect the results. There are no criteria for finding an efficient value for *α* either.

CC also receives as an input parameter the number of biclusters to obtain, since it is based on a sequential covering strategy, as well as Evo-Bexpa.

OPSM approach is based on the formulation of a probabilistic model of the expression data. As finding the best model is infeasible for real data, Ben-Dor et al. use partial models and grow them iteratively. The algorithm takes as input paramater the number of partial models passed for each iteration *ℓ*. According to the authors, increasing *ℓ* would improve results, although it will come at a cost of a higher running time. Nevertheless, it is no clear in which aspect does the modification of *ℓ* affect the obtained biclusters (size, quality or other) in real data. Furthermore, they do not provide any instruction on how to select an appropriate value for *ℓ*.

The Iterative Signature Algorithm (ISA) receives three different input parameters. *T*_*g*_ and *T*_*c*_ are thresholds for the resolution of the modular decomposition of both genes and conditions, respectively. *T*_*c*_ is said to have a minor effect on the results, and was set to 2 in all the analyses. *T*_*g*_ was varied from 1.8 to 4.0 in steps of 0.1, in order to analyse the resulting stringency of co-regulation between the genes. The default value for *T*_*g*_ can be assumed as 2.0. Although the authors perform an analysis on the influence of *T*_*g*_ on the results on a specific dataset[36], it is not straightforward to see what will the influence be for any other datasets.

The third input parameter for ISA is the number of starting points that the algorithm uses for randomly selecting a set of genes and iteratively refining this set until the genes and conditions in it are mutually consistent and match the definition of a transcription module. Authors claim that using a sufficiently large number of initial sets it is possible to determine all the modules corresponding to a particular pair of thresholds. The default value for this parameter is set to 100.

xMOTIFs looks for biclusters in which genes are expressed in the same state across all samples. In order to differentiate biologically interesting states, a maximum p-value parameter is used, considering only those states whose p-value is less than the parameter (1×10*E*−9). Another parameter *α* determines the minimum number of samples for biclusters, given as a fraction of the total number of conditions, being its default value 0.05. Murali and Kasif also make use of inner parameters to the algorithm such as the number of seeds (*n*_*s*_), the number of determinants (*n*_*d*_) and the size of the discriminating set (*s*_*d*_), as in [56]. The authors claim that the quality of the results does not change much when those are slightly varied.

#### Analysis of Evo-Bexpa parameter sensibility on real dataSets

Input parameters for Evo-Bexpa were detailed in section *Methods*. The number of parameters will depend on the number of objectives or bicluster characteristics to optimize. In this approach, we have used 5 different configuration parameters, which control the volume (*w*_*g*_, *w*_*c*_ and *w*_*s*_), the amount of overlapping (*w*_*ov*_) and the gene variance (*w*_*var*_).

Default values for Evo-Bexpa have been set experimentally by using a benchmark database and trying to reproduce previous results for this database in the literature. Also, solutions with low proportions of the number of genes and high percentage of the total number of samples has been favoured for the setting.

In order to deduce the parameter influence on each characteristic, we have tested Evo-Bexpa modifying each configuration parameter from −100*%* to +100*%* its value, in intervals of ±25*%*. Table 1 shows all the used values, where the central row gives the default ones. So it means running Evo-Bexpa 8 additional times per parameter, using each value of Table 1 for each weight, while maintaining the other weights at their default values, this represents 41 experiments for each dataset. Furthermore, we have chosen four different microarrays to study the parameters influence in diverse scenarios (see Table 1). All in all, for the purpose of the parameter influence analysis, a total of 164 experiments over real datasets have been carried out, being 100 the number of biclusters to be obtained in each execution. All weights must have a positive value, being 0.0 the value for which the corresponding objective exerts no influence on the results. However, they can be set to any positive value, even above +100*%* their default values, if more influence of any bicluster characteristic is desired.

**Table 1:** Experimental values for configuration parameters

	***w***_***g***_	***w***_***c***_	***w***_***s***_	***w***_***ov***_	***w***_***var***_
−100*%*	0.0	0.0	0.0	0.0	0.0
−75*%*	0.0625	0.125	1.25	1.25	0.025
−50*%*	0.125	0.25	2.5	2.5	0.05
−25*%*	0.1875	0.375	3.75	3.75	0.075
Default	0.25	0.5	5.0	5.0	0.1
+25*%*	0.3125	0.625	6.25	6.25	0.125
+50*%*	0.375	0.75	7.5	7.5	0.15
+75*%*	0.4375	0.875	8.75	8.75	0.175
+100*%*	0.5	1.0	10.0	10.0	0.2

This table shows the different configuration parameter values used in our experimentation. Each weight has been modified from −100*%* to +100*%* its default value, in intervals of ±25*%*. Default values in the central row have been obtained with synthetic data experiments. Variations have been made individually, invoking Evo-Bexpa for each different weight value, while maintaining the other weights at their default values.

In the following, parameter analysis is only presented for *Embryonal tumours of the central nervous system* dataset [57], in view of results for the other datasets are similar and do not contribute anything new to the study.

**Figure 3:**
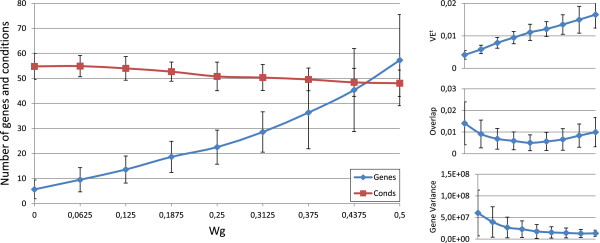
***w***_***g***_** influence over the different bicluster features.** Main graphic shows the variations of the means and deviations of both the number of genes and conditions, depending on the values of *w*_*g*_ in abscissa axis. At the right side of the main graphic, the way in which the variations of *w*_*g*_ affects VE ^*t*^, overlap and mean gene variance has also been represented.

**Figure 4:**
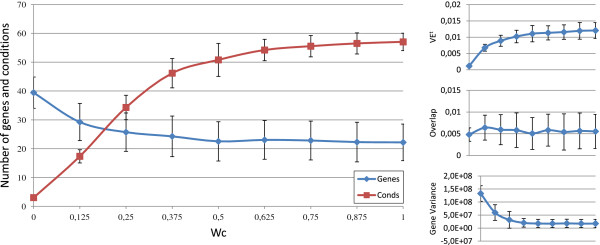
***w***_***c***_** influence over the different bicluster features.** Main graphic shows the variations of the means and deviations of both the number of genes and conditions, depending on the values of *w*_*c*_ in abscissa axis. At the right side of the main graphic, the way in which the variations of *w*_*c*_ affects VE ^*t*^, overlap and mean gene variance has also been represented.

**Figure 5:**
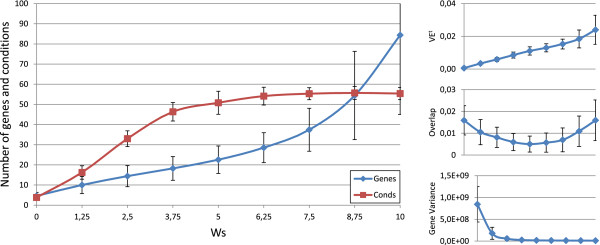
***w***_***s***_** influence over the different bicluster features.** Main graphic shows the variations of the means and deviations of both the number of genes and conditions, depending on the values of *w*_*s*_ in abscissa axis. At the right side of the main graphic, the way in which the variations of *w*_*s*_ affects VE ^*t*^, overlap and mean gene variance has also been represented.

**Figure 6:**
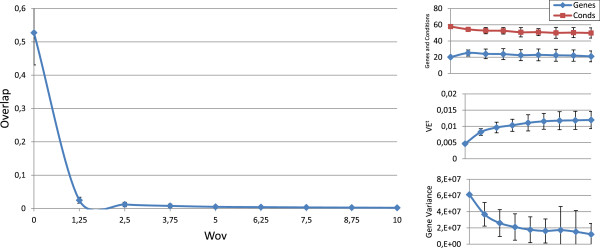
***w***_***ov***_** influence over the different bicluster features.** Main graphic shows the variations of the means and deviations of the overlap term in equation 9, depending on the values of *w*_*ov*_ in abscissa axis. At the right side of the main graphic, the way in which the variations of *w*_*ov*_ affects the number of genes and conditions, VE ^*t*^ and mean gene variance has also been represented.

**Figure 7:**
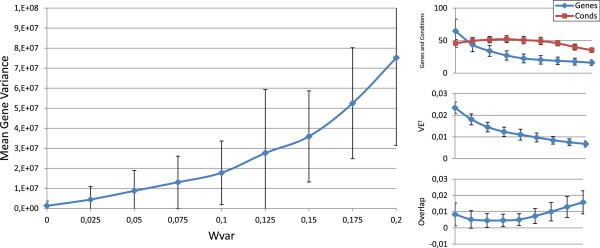
***w***_***var***_** influence over the different bicluster features.** Main graphic shows the variations of the means and deviations of the mean gene variance term in equation 10, depending on the values of *w*_*var*_ in abscissa axis. At the right side of the main graphic, the way in which the variations of *w*_*var*_ affects the number of genes and conditions, VE ^*t*^ and the overlap term has also been represented.

Figures 3, 4, 5, 6 and 7 represent the variations of the means and deviations for all the different objectives (VE ^*t*^, number of genes, number of conditions, overlap and gene variance) when modifying each configuration parameter. Each figure is made up of four different graphics which depict the influence of a certain weight over the aforementioned bicluster aspects. The main graphic of each figure shows the variations of the means and deviations for the main aspect affected by the weight modifications. Abscissa axis refers to the specific weight values, according to Table 1, while the ordinates axis depends on the configuration parameter under study. For example, in the first three Figures (3, 4 and 5), vertical axis corresponds to the means of the number of elements in the biclusters (genes or samples). At the right side of the main graphic, the way in which the variations of the parameter affects the other characteristics has also been represented.

Means of the 100 biclusters represent the general tendency of the results. Nevertheless, deviations cannot be disregarded. This is due to the fact that although it is possible to favour some properties in the solutions, results provided by Evo-Bexpa are diverse, obtaining thus biclusters in which their properties vary in a range around the reported mean.

Although the modification of any configuration parameter not only affects its corresponding aspect, it can be clearly seen that the greatest variations in any characteristic are obtained by increasing or decreasing its associated weight. Furthermore, some objectives are related in a negative way. Mean gene variance, for instance, would be decreased if bicluster size is increased or the overlap decreases. Therefore, it would be a good practice to slightly correct gene variance parameter when size or overlap parameters are adjusted, or vice-versa. Other characteristics have different behaviours when adjusting any other weights. The mean of the number of genes and conditions is quite stable when modifying *w*_*ov*_ in Figure 6, as well as overlap mean when *w*_*c*_ is adjusted, in Figure 4. In general, VE ^*t*^ increases whenever greater sizes or less overlapping is preferred. It was the expected behaviour, since bigger solutions would produce higher values of VE ^*t*^, unless they were closer to a perfect combined pattern. On the contrary, biclusters with higher mean gene variance would have lower values of VE ^*t*^, due to the reduction of their sizes when higher variances are required.

Table 2 presents a summary of the configuration parameters influences over the different bicluster characteristics. This table has been elaborated using the four real datasets in Table 3 and the aforementioned variations of the weights. This way, Table 2 represents the common behaviour observed in all the datasets under study.

**Table 2:** Qualitative influence of the configuration parameters over the different objectives

**Weight**	***VE***^***t***^	**#Genes**	**#Conditions**	**Overlap**	**Mean gene variance**
*w*_*g*_	⇈	⇈	=	↘↗	⇊
*w*_*c*_	*↑*	↘=	⇈	=	⇊
*w*_*s*_	⇈	⇈	⇈	↘↗	⇊
*w*_*ov*_	*↑*	=	=	⇊	⇊
*w*_*var*_	⇊	⇊	↗↘	↘↗	⇈

Each row represents the influence of each configuration parameter in the first column over the characteristics in the first row, where the behaviour of each row has been observed when increasing the corresponding weight from 0.0 to its maximum experimented value (see Table 1). Symbol ⇈ represents large increments, *↑* medium increments and ⇊ large decrements. Symbol = stands for no significant variations, ↘↗ depicts a decreasing behaviour for low values of the weight, turning to increasing for higher values of the parameter, while ↗↘ depicts the contrary situation.

**Table 3:** Datasets used in the experimentation

**Dataset**	**Name**	**#Genes**	**#Conditions**	**Ref.**
Yeast	Yeast *Saccharomyces cerevisiae* cell cycle	2884	17	[58]
Embryonal	Embryonal tumors of the central nervous syst.	7129	60	[57]
Leukemia	Leukemia	7129	72	[4]
Steminal	Steminal Cells	26127	30	[59]

This table includes the name, sizes and references to the corresponding publications of the four datasets used in our experimentation. Yeast dataset represents one the most used dataset for comparison of biclustering techniques, and is considered to be a benchmark dataset.

In short, Evo-Bexpa parametrization allows the user to specify preferences on biclusters features, by adjusting the corresponding weight(s). The recommended procedure consist in first run the algorithm using the default configuration, correcting afterwards those weights needed to reach the desired results in terms of the objectives. In order to select an appropriate correction, Figures 3, 4, 5, 6 and 7, together with the information in Table 2 should be used, being aware of the implications that each weight variation has on the other bicluster aspects.

### Synthetic data experiments

In order to test the effectiveness of Evo-Bexpa to find biclusters following shifting and scaling patterns, we have carried out a set of experiments inspired on the works of A. Mukhopadhyay et. al [30] and D. Bozdag et. al [22], where perfect synthetic biclusters with shifting or scaling tendencies were inserted into artificial data sets. In a more general purpose, we have used combined patterns (shifting and scaling simultaneously) for biclusters generation. These biclusters have been hidden in several artificial data matrices, with uniform random distributions.

We have chosen the size of one of the most tested benchmark microarrays in biclustering: yeast *Saccharomyces cerevisiae cell cycle expression* dataset [58], made up of 2884 genes and 17 samples, for the generation of artificial matrices. We have also defined several sizes (genes × conditions) for the inclusion of perfect biclusters: 20×10, 60×12, 100×13, 150×15 and 200×16. For each of these sizes we have generated a perfect bicluster according to a combined shifting and scaling pattern. Each of these 5 different sized perfect biclusters has been inserted into 5 different random in silico microarrays in random positions. Thus, a total of 25 different case studies constitute the first set of experiments, in which no noise has been introduced.

Furthermore, we have also generated the same number of experiments adding noise to the data with random values generated from normal distribution, with mean equals to 0 and deviation equals to 0.25. All in all, there are 50 different experiments, 25 in which the biclusters follow a perfect pattern and 25 in which random noise has been included into the data.

For each of the experiments, we have run the following 6 different biclustering algorithms: OPSM, ISA, xMotifs, CC, Bimax and Evo-Bexpa presented in this work. We have used default parameters to run all of them.

In order to check the extent to which the bicluster obtained by each algorithm adjusts to the solution we have used match scores indexes for both genes and conditions [38] as performance measure. Let ${ℬ}_{1}(I_{1},J_{1})$ and ${ℬ}_{2}(I_{2},J_{2})$ be two biclusters, then gene match score is defined as $S_{I}(I_{1},I_{2})=\frac{\left|I_{1}\cap I_{2}\right|}{\left|I_{1}\cup I_{2}\right|}$ and condition match score is defined as $S_{J}(J_{1},J_{2})=\frac{\left|J_{1}\cap J_{2}\right|}{\left|J_{1}\cup J_{2}\right|}$. Both indexes vary from 0, when both set of genes (or conditions) are disjoint, to 1, when the sets totally match. This way, match score indexes can be use to compute the degree of similarity of the sets of genes and conditions of two biclusters. We have, therefore, compare each bicluster obtained with the corresponding solution for all the executions using the six former algorithms.

**Figure 8:**
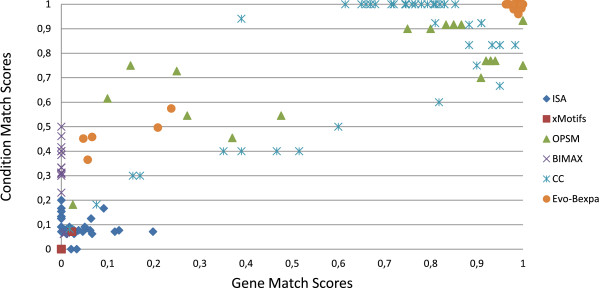
**Gene and condition match scores for ISA, xMotifs, OPSM, BIMAX, CC and Evo-Bexpa in synthetic experiments.** X-axis represents gene match scores and Y-axis represents condition match scores. Each dot in the graphic refers to the comparison of a bicluster found by each algorithm and its equivalent solution. Dots in the right top corner of the graphic correspond to those obtained biclusters which have a better match with its equivalent solution.

Figure 8 displays the gene and condition match scores of the executions of the six algorithms. X-axis represents gene match scores and Y-axis represents condition match scores. Each dot in the graphic refers the comparison of a bicluster found by each algorithm and the equivalent solution. According to the gene and condition match scores definitions, the dots in the right top corner of the graphic correspond to those obtained biclusters which have a better match with its equivalent solution. OPSM, CC and Evo-Bexpa are the algorithms with better results. In the case of Evo-Bexpa, there exists exactly five biclusters which are not correctly found, and whose scores indexes are below 0.6 and 0.3 for the conditions and genes sets respectively. We have studied these results and have found that they correspond to the five experiments in which the hidden biclusters are smaller (20×10) and noise has been introduced. Only OPSM finds better solutions than Evo-Bexpa in these experiments, while for the other cases Evo-Bexpa outperforms both OPSM and CC. In fact, we have conducted a statistical test which confirms that Evo-Bexpa outperforms the other five algorithms in finding perfect shifting and scaling behaviours in synthetic data.

Match Score can also be used for measuring the degree of similarity of two biclusters using former genes and conditions match scores indexes. This way, we have used the bicluster match score index in order to rank the effectiveness of the algorithms. Bicluster match score is defined as $\sqrt{S_{I}(I_{1},I_{2})\times S_{J}(J_{1},J_{2})}$, and varies from 0, when the biclusters ${ℬ}_{1}$ and ${ℬ}_{2}$ are disjoints, to 1, when ${ℬ}_{1}$ and ${ℬ}_{2}$ completely match.

Since our results do not follow a normal distribution, we have applied Friedman as a non-parametrical test to carry out a comparison which involves six different methods. Friedman test ensures us that the results obtained by the six algorithms are statistically different, with a p-value of 1.16^−10^. Also, the raking provided by Friedman suggests the following order: Evo-Bexpa, OPSM, CC, ISA, Bimax, xMotifs, which seems to be in concordance with the representation in Figure 8. Furthermore, we have also performed a post-hoc procedure in order stablish a comparison two by two using our algorithm as the control method. In this comparison, we obtained for each of the other five algorithms a p-value less than the alpha values returned by five different post-hoc procedures (Holm, Holland, Rom, Finner and Li), which certifies that our proposal Evo-Bexpa outperforms the other five algorithms in this empirical study with a significance less than 0.05. STATService [60] has been used in order to perform these statistical tests.

### Experiments on real DataSets

Experiments on four different real microarrays have been conducted using Evo-Bexpa and the five algorithms contained in Bicat toolbox: OPSM, CC, ISA, Bimax and xMotifs. Table 3 specifies the details of the datasets, including theirs sizes as well as references to their corresponding publications. Yeast dataset is the smallest, made up of 2884 genes and 17 samples, and represents one the most used dataset for comparison of biclustering techniques. In fact, it is considered as a benchmark dataset for many researches. Leukemia dataset is the one containing the higher number of samples, while Steminal acts as the most unbalanced microarray, with the mayor number of genes (26127) and only 30 samples.

Table 4 presents the results for each dataset and algorithm using default parameters in all cases. Results are represented by the number of biclusters obtained, means and deviations of their volume (number of genes and experimental conditions), and means and deviations of their VE ^*t*^ values and gene variance (see section *Methods*). Results have also been grouped by dataset, being the first five rows those corresponding to the executions of OPSM, ISA, CC, Bimax and Evo-Bexpa for Yeast microarray, respectively. Unfortunately, Bicat implementations of xMotifs and Bimax approaches did not work properly for every dataset in Table 3. Specifically, xMotifs could not be performed for Yeast and Steminal datasets, due to unexpected runtime errors. xMotifs could neither be executed for Leukemia dataset, since it does not support more than 64 samples, according to Bicat toolbox. In the case of Bimax, we did not obtain results for either Embryonal, Leukemia or Steminal datasets in reasonable time. This fact might be related to the datasets sizes, since the mean of the biclusters sizes for the Yeast dataset using Bimax is greater than 75% of the size of the whole microarray, and the computational cost of generating quality biclusters of similar proportions for the other datasets may be unfeasible. Nevertheless, Bimax has run properly for the Yeast dataset and synthetic data matrices of the same size.

**Table 4:** **Summary of experimental results for the microarrays in Table** 3

**Dataset**	**Algorithm**	**NumBic**	**Genes**	**Conditions**	**VEt**	**Mean gene variance**	
	OPSM	14	496.1 ± 791.1	8.6 ± 4.4	0.189 ± 0.051	1.39×10^5^ ± 2.23×10^5^	
	ISA	0	-	-	-	-	
Yeast 2884x17	CC	100	34.0 ± 64.2	7.6 ± 3.2	0.151 ± 0.048	3.20×10^3^ ± 3.16×10^3^	
	Bimax	56	2297.6 ± 26.1	15.3 ± 0.5	0.207 ± 0.004	1.42×10^5^ ± 5.41×10^3^	
	Evo-Bexpa	100	44.0 ± 33.7	11.8 ± 3.9	0.051 ± 0.027	9.81×10^2^ ± 5.00×10^2^	
	OPSM	12	1151.5 ± 1809.1	7.8 ± 4.1	0.155 ± 0.072	2.51×10^8^ ± 4.22×10^8^	
	ISA	20	377.0 ± 191.0	2.7 ± 0.8	0.145 ± 0.053	5.98×10^8^ ± 3.85×10^8^	
ET 7129x60	xMotifs	1000	2134.6 ± 830.4	5.0 ± 0.0	0.135 ± 0.021	9.61×10^7^ ± 3.74×10^7^	
	CC	100	53.1 ± 100.4	11.9 ± 7.9	0.310 ± 0.082	6.14×10^4^ ± 2.06×10^5^	
	Evo-Bexpa	100	22.5 ± 6.8	50.8 ± 5.7	0.011 ± 0.003	1.78×10^7^ ± 1.59×10^7^	
	OPSM	12	924.3 ± 1633.3	7.8 ± 4.3	0.103 ± 0.047	1.75×10^8^ ± 2.89×10^8^	
Leukemia 7129x72	ISA	34	253.1 ± 172.1	3.1 ± 1.1	0.147 ± 0.049	3.31×10^8^ ± 2.17×10^8^	
	CC	100	53.5 ± 232.4	13.9 ± 8.6	0.265 ± 0.067	4.32×10^4^ ± 8.53×10^4^	
	Evo-Bexpa	100	18.4 ± 2.9	63.3 ± 5.9	0.008 ± 0.002	4.46×10^6^ ± 2.97×10^6^	
	OPSM	27	1170.6 ± 3274.1	16.2 ± 8.8	0.399 ± 0.163	1.73×10^7^ ± 5.55×10^7^	
Steminal 26127x30	ISA	0	-	-	-	-	
	CC	100	179.1 ± 813.6	13.0 ± 3.1	0.219 ± 0.071	4.84×10^4^ ± 1.19×10^5^	
	Evo-Bexpa	100	33.8 ± 17.9	26.3 ± 2.4	0.009 ± 0.004	4.80×10^5^ ± 2.69×10^5^	

Results are represented by the number of biclusters obtained, means and deviations of their volume (number of genes and experimental conditions), and means and deviations of their VE ^*t*^ values and mean gene variance (see section *Methods*).

Bimax generated 56 biclusters for Yeast dataset, all of them of a very big size, containing almost the totality of the elements, both genes and conditions. In fact, although we did not measure overlap for the algorithms in Bicat, it must be certainly high, since biclusters are made up of a mean of almost 2300 genes (out of 2884) and 15,16 or 17 experimental conditions (out of 17). Studying correlations in this kind of biclusters is almost as difficult as studying the whole dataset. It would even be easier to analyse the genes and/or samples not contained in the biclusters.

Murali and Kasif’s xMotifs generates 1000 biclusters for the Embryonal Tumours dataset, all of them have 5 samples and a decreasing number of genes with the biclusters indexes. The first bicluster is made up of 4593 genes, more than the half of the whole dataset, while bicluster number 999 consist of 576 genes. We consider the number of biclusters to be cumbersome for any post analysis, even more if it needs to be carried out manually. Also, the number of genes per bicluster may again result too high for any specific study.

Iterative Signature Algorithm (ISA) only found biclusters for Embryonal Tumours (20) and Leukemia (12) microarrays. In both cases they are obtained with a decreasing number of genes and conditions, being the second one a very low value, which we consider almost useless in biclustering analyses (2 or 3 samples per bicluster). The number of genes varies from 661 to 81 for the Embryonal database and from 707 to 83 for Leukemia. For both datasets the biclusters obtained by ISA have the greatest gene variance.

OPSM, together with CC and Evo-Bexpa produced results for the four datasets. OPSM biclusters are characterized for having the greatest deviation on the number of genes. In fact, OPSM bicluster’s sizes vary from a bicluster containing a few genes and a great number of samples to the contrary: almost the whole set of genes and very few samples (2 ×17 to 2422 ×2 for Yeast, 2 ×16 to 5491 ×2 for ET, 2 ×17 to 5208 ×2 for Leukmia and 6 ×30 to 15332 ×2 in the Steminal case). From the biological point of view, only a small portion of the obtained biclusters are interesting: those in the intermediate situations.

CC algorithm allows the user to choose the number of biclusters to obtain, being 100 its default value. It is a sequential process in which random data is inserted into the matrix. For these reasons, first biclusters are in general greater than the following ones, being the smallest ones the last 10 biclusters. VE ^*t*^ values are quite high, specially for Embryonal Tumours ($\bar{VE^{t}}=0.3098$) and Leukemia ($\bar{VE^{t}}=0.2652$) datasets, where biclusters sizes are not as big as to favour this range of values. Also, results produced by CC are rather flat, since their gene variance is in the majority of the cases the lowest of all the algorithms.

Default parameters for Evo-Bexpa (Table 1) have been adjusted to produce biclusters with a very low proportion of genes but a high proportion of samples, although there exists considerable diversity in the results, as shown by the deviation. Only for the Yeast dataset Evo-Bexpa obtains the biclusters with the lowest values of gene variance, while VE ^*t*^ is always much lower, as preferred. In fact, VE ^*t*^ values for the biclusters found by Evo-Bexpa is smaller than 0.1, for all datasets, whereas no other algorithm finds biclusters with such a low VE ^*t*^ level. This is a very good achievement of our approach given the importance of VE ^*t*^ as a quality measure for quantifying all kind of patterns in gene expression data (see section *Methods*). Furthermore, although VE ^*t*^ values increase for bigger biclusters or those with lower levels of overlapping, it can be seen in Figures 1, 3, 4, 5 and 6 that they are never greater than the biclusters VE ^*t*^ for the other approaches. The order in which biclusters are found with Evo-Bexpa is not relevant, although if the weights associated to the overlapping and size are too high Evo-Bexpa will produce big submatrices with no overlap, increasing thus VE ^*t*^ considerably for the latest solutions.

The great advantage of Evo-Bexpa with regard to the other algorithms is its ability to adjust the result characteristics to user defined parameters. Next subsection presents biological validation for biclusters obtained by Evo-Bexpa, using the same parameter configuration introduced in section *Analysis of Evo-Bexpa Parameter Sensibility on Real DataSets*, which confirms the validity of our approach.

#### Biological assessment

The Gene Ontology project [61] (GO) is a initiative to unify the representation of gene and gene product attributes across all species. It is a directed acyclic graph whose nodes represent terms dealing with molecular functions, cell components or biological processes, and edges connecting nodes depict dependency relationships. Gene Ontology has been widely used in genome research applications, and also for the validation of results obtained after a microarray analysis process, such as clustering or biclustering.

Term-for-Term analysis represents the standard method of performing statistical analysis for over-representation in GO. Starting from a subset of genes (study group) from a larger population (whole set of genes in the microarray), we are interested in knowing if the frequency of an annotation to a Gene Ontology term is relevant for the study group compared to the overall population. Fisher’s exact test is the most commonly used test for this purpose, together with the Bonferroni multiple test correction. This correction is advisable to be performed since Fisher’s test is applied to many terms per study group. After that, a Bonferroni adjusted p-value is obtained for each GO term for which genes in the study group are involved. In our case, study groups correspond to the sets of genes in each bicluster. Depending on the desired confidence level, which determines the adjusted p-value, a bicluster is said to be significantly enriched if there exists at least one GO term for which genes in the bicluster are significantly annotated.

Among all the existing tools for the analysis of gene expression data using GO [62] we have chosen Ontologizer [63] for assessing Evo-Bexpa biclusters due to its novelty (it has been recently updated) and its suitability for performing the validation of a great number of biclusters as a batch process.

Results of bicluster biological validation using GO vary depending on the biclusters sizes. In fact, GO terms are organized in levels of the graph according, among other issues, to their specificity [64]. Terms in higher levels (nearer to the root of the graph) are considered to be more generic and have a greater number of genes annotated, while terms in lower levels of the graph are more specific and might have only a few genes annotated. For this reasons, when working with big sets of genes, it would be more probable that they will be enriched for more generic GO terms (higher in the graph structure).

**Figure 9:**
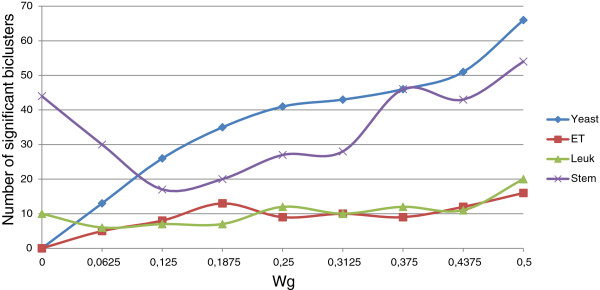
**Number of significant biclusters for different*w***_***g***_** values.** Ordinates axis represents the number of significant biclusters obtained for each dataset and the *w*_*g*_ values in abscissa axis, being the adjusted p-value 0.05.

**Figure 10:**
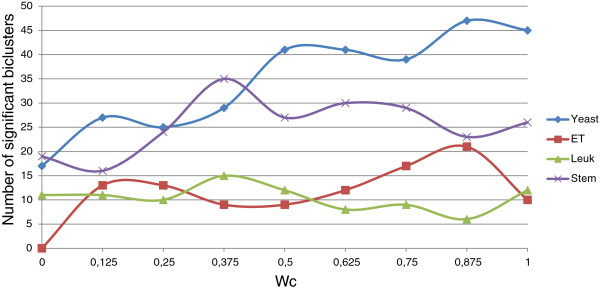
**Number of significant biclusters for different*w***_***c***_** values.** Ordinates axis represents the number of significant biclusters obtained for each dataset and the *w*_*c*_ values in abscissa axis, being the adjusted p-value 0.05.

**Figure 11:**
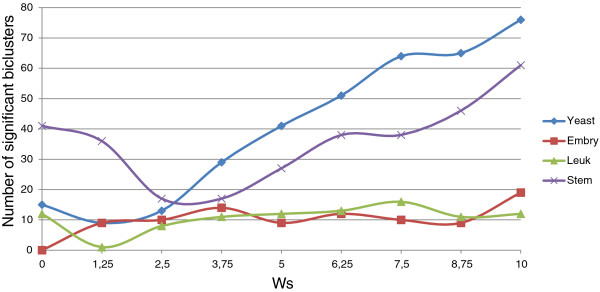
**Number of significant biclusters for different*w***_***s***_** values.** Ordinates axis represents the number of significant biclusters obtained for each dataset and the *w*_*s*_ values in abscissa axis, being the adjusted p-value 0.05.

**Figure 12:**
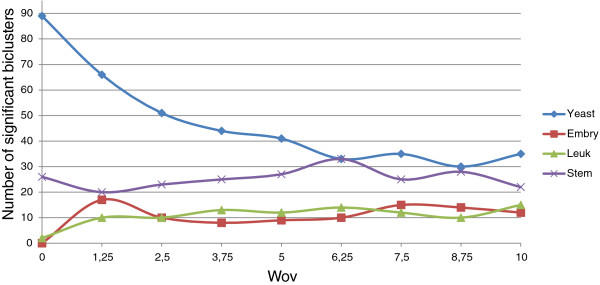
**Number of significant biclusters for different*w***_***ov***_** values.** Ordinates axis represents the number of significant biclusters obtained for each dataset and the *w*_*ov*_ values in abscissa axis, being the adjusted p-value 0.05.

**Figure 13:**
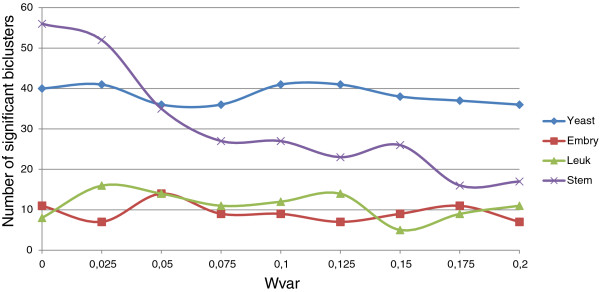
**Number of significant biclusters for different*w***_***var***_** values.** Ordinates axis represents the number of significant biclusters obtained for each dataset and the *w*_*var*_ values in abscissa axis, being the adjusted p-value 0.05.

In order to check the influence of Evo-Bexpa configuration parameters on the biological validation of the obtained biclusters, we have represented in Figures 9, 10, 11, 12 and 13 the number of significant biclusters (ordinates axis) for each of the experiments detailed in section *Analysis of Evo-Bexpa Parameter Sensibility on Real DataSets* (see Table 1) and for each dataset, where abscissa axis refers to the specific weight value, and the adjusted p-value has been set to 0.05.

The main conclusion we can come up to is that there is no a common behaviour embraced by the four different data sets and for each configuration parameter. For example, the number of significant biclusters for the Yeast dataset increases significantly whenever the number of genes (*w*_*g*_) or conditions (*w*_*c*_) are increased, as well as the overall size (*w*_*s*_). Nevertheless, when the overlap gets more penalized (Figure 12), the number of significant biclusters for the Yeast dataset decreases. This is due to the fact that biclusters sizes are affected by the overlapping weight, in the reverse way (the more restrictive the overlapping amount is, the less elements the biclusters contains). Figure 13 shows that variance weight variations do not significantly affect the number of significant biclusters in the Yeast dataset.

Steminal dataset is the second one presenting more variations on the number of significant biclusters when modifying the parameters values. It is worth to note that Steminal is the only dataset for which the number of significant biclusters varies significantly from more to less when increasing the mean gene variance. This is related to the fact that when higher gene variances in biclusters are required, the size of the obtained biclusters decrease, as explained in *Analysis of Evo-Bexpa Parameter Sensibility on Real DataSets* section.

For Embryonal and Leukemia data sets the number of significant biclusters is quite lower than for the other two data sets, in all the cases. In fact, it rarely exceeds 20%. For both of them there no exist great variations when modifying the different parameter values. It is interesting to mark that no significant biclusters were found for the Embryonal datasets when *w*_*g*_,*w*_*c*_ or *w*_*s*_ are set to zero (Figures 9, 10 and 11).

One common issue in hierarchical ontologies is deciding the level of specificity to use in the analysis [65]. On the one hand, GO terms that are too general may overlook significantly represented biological markers because many genes in the background genome are also annotated by the general GO terms. On the other hand, GO terms that are too specific can result in the same problem, since too few genes in the microarray are annotated by these GO terms. In order to study the level of specificity of the terms to which Evo-Bexpa biclusters have been annotated we have carried out three different validations: taking the whole hierarchical graph into account, and limiting the validation with the levels 3 to 6 and 4 to 7, both inclusive.

Table 5 presents the validation results for Evo-Bexpa biclusters using the default configuration. For each type of validation the number of significant biclusters and the mean of their significant terms are given, for two different adjusted p-value values: 0.01 and 0.05. As it can be seen in Table 5, the number of significant biclusters slightly varies from the validation with the whole graph to the limited validations, meaning that the majority of biclusters obtained by Evo-Bexpa contain genes that are not frequently annotated to too general or too specific terms. For the Embryonal Tumours dataset the number of significant biclusters does not even decrease with the limited validations. The mean of significant terms to which genes in the biclusters of the previous column have been annotated is also smaller for the hierarchically limited validations. This was an expected result since those terms which are not in the specified levels have not been taken into account in the study. Nevertheless, we consider the reduction on the number of significant biclusters and terms to be minimal, locating Evo-Bexpa biclusters in the central part of the GO graph.

**Table 5:** Validation results with GO hierarchy level limitations

		**All levels**		**Levels 3 to 6**		**Levels 4 to 7**	
	**p-value**	***#*Bics**	$\bar{\mathrm{\#Terms}}$	***#*Bics**	$\bar{\mathrm{\#Terms}}$	***#*Bics**	$\bar{\mathrm{\#Terms}}$
Yeast	0.01	32	5.969	32	5.125	31	4.387
	0.05	41	6.878	40	5.625	40	5.225
ET	0.01	3	3.000	3	3.000	3	2.666
	0.05	9	3.778	9	3.444	9	3.333
Leukemia	0.01	4	1.000	3	1.000	3	1.000
	0.05	12	2.333	11	1.454	11	1.818
Steminal	0.01	18	4.056	16	2.750	13	2.231
	0.05	27	7.111	26	5.269	25	4.200

Validation with Gene Ontology has been carried out for Evo-Bexpa biclusters of the four datasets in Table 3 using the default configuration. This table shows the number of significant biclusters and significant terms in three different validations: taking the whole hierarchical graph into account, and limiting the validation with the levels 3 to 6 and 4 to 7, both inclusive.

In general, the validation carried out support Evo-Bexpa effectiveness for biclustering microarray data. In fact, significant biclusters have been obtained for each dataset at 0.01 and 0.05 levels provided that configuration parameters are not set to zero. This fact also suggest the appropriateness of the different chosen objectives in this work. Although the number of significant biclusters may vary in a different way for different datasets when modifying the different configuration parameters, Evo-Bexpa significant biclusters correspond to significant terms in the central part of the GO graph. This means Evo-Bexpa succeeds at finding biclusters whose significant terms have an intermediate level of specificity.

## Conclusion

In this paper we have presented a new evolutionary algorithm for biclustering of gene expression data named Evo-Bexpa. There exist two main advantages over other existing approaches: the use of an evaluation measure able to detect shifting and scaling patterns (VE ^*t*^), and the possibility of specifying user preferences on some characteristics of the results (number of genes and conditions, overlapping amount,...). This way, if any previous information related to the microarray under study is available, the search can be guided towards the preferred types of biclusters. Furthermore, other objectives can also be easily incorporated into the search, as well as any objective may be ignored by setting its weight to zero. Default values for the configuration parameters are given in order to provide the user with quality results. Moreover, an experimental study has been performed on four real datasets in order to study the parameters sensibility and their influence over the different features. This study concludes with an useful guide on how to customize the algorithm depending on the user preferences.

Experimental results on both synthetic and real datasets confirm the validity of our approach, where the results have been compared to those obtained by five well-known biclustering algorithms. Evo-Bexpa has been proven to outperform ISA, xMotifs, OPSM, BIMAX and CC in synthetic experiments, where match scores indexes have been used for comparing the obtained results with the solution. Regarding the experiments on real datasets, Evo-Bexpa results have been biologically validated using different levels in Gene Ontology hierarchy. This validation shows that significant biclusters obtained by Evo-Bexpa correspond to neither too general or specific GO terms.

## Endnote

^a^Biclustering Analysis Toolbox (BicAT) is a software platform for clustering-based data analysis that integrates various biclustering and clustering techniques in terms of a common graphical user interface [39]. It has been used in this work for the comparison of Evo-Bexpa performance with ISA, xMotifs, OPSM, BIMAX and CC algorithms and is publicly available at http://www.tik.ee.ethz.ch/sop/bicat/.

## Competing interests

The authors declare that they have no competing interests.

## Authors’ contributions

BP, RG and JSAR conceived the approach. Implementation of the method and performing the computational experiments were done by BP. All authors read and approved the final manuscript.
